# Case report: Tissue positivity for SARS-CoV-2 in a preterm born infant death of thrombosis: possible intrauterine transmission

**DOI:** 10.3389/fmed.2023.1127529

**Published:** 2023-05-11

**Authors:** Salvatore Greco, Juana Maria Sanz, Daria Bortolotti, Chiara Marina Semprini, Carlotta Braga, Roberta Gafà, Erica Santi, Iva Maestri, Roberta Rizzo, Pantaleo Greco, Angelina Passaro

**Affiliations:** ^1^Department of Translational Medicine, University of Ferrara, Ferrara, Italy; ^2^Department of Chemical, Pharmaceutical and Agricultural Sciences, University of Ferrara, Ferrara, Italy; ^3^Obstetrics and Gynecology Unit, Department of Medical Sciences, University of Ferrara, Ferrara, Italy; ^4^Research and Innovation Section, University Hospital of Ferrara Arcispedale Sant’Anna, Ferrara, Italy

**Keywords:** SARS-CoV-2, intrauterine transmission, newborn, thrombosis, gastrointestinal tract, pulmonary embolism, placenta

## Abstract

Intrauterine transmission of SARS-CoV-2 (Severe Acute Respiratory Syndrome Corona Virus 2) is still matter of debate among scientists and there is limited information concerning this aspect of research. This could lead to severe complications of the growing fetus and, theoretically, of the newborn as well. We report the case of a male infant of 1,100 grams, born at 27th week of gestation to a SARS-CoV-2 mother, tested negative for viral detection at delivery. He was immediately admitted to neonatal Intensive Care Unit (ICU) for severe complications, where he died after 37 days by pulmonary embolism and thrombosis of the superior vena cava. After autopsy, SARS-CoV-2 N-protein and Spike RBD were detected in several tissues, particularly in the esophagus, stomach, spleen, and heart, with a significantly higher H-Score than the placenta. In conclusion, immunohistochemical analysis demonstrated SARS-CoV-2 NP and Spike RBD positivity in different tissues suggesting a possible intrauterine transmission. Newborn thrombo-embolism could be a complication of SARS-CoV-2 infection as observed in adult patients.

## Introduction

COVID-19 (Corona Virus Disease 2019), the clinical expression of SARS-CoV-2 infection, might include systemic inflammation and pro-coagulative processes. The rate of thromboembolic events (TE) related to SARS-CoV-2 (Severe Acute Respiratory Syndrome Corona Virus 2) was estimated to range from 25 to 30% in the critically ill population (1) and some researchers from Italy (2) recently reported the occurrence of pediatric venous TE events, arterial thrombosis or intracardiac thrombosis (54, 38, and 8%, respectively), which increased the risk of death among COVID-19 children from 1% (without TE complications) to 12–28%.

Among the fragile populations, pregnant women seem to be predisposed to develop severe COVID-19 with frequent admissions to the Intensive Care Units (ICUs). Fortunately, the rate of mother-to-newborn transmission is less than 5% (3) and neonatal deaths represent a rare event (4).

Examination of placental samples from SARS-CoV-2 nasopharyngeal swab-positive mothers showed differences in viral positivity, and most of the newborns tested negative without virus-induced diseases. Nevertheless, some cases of SARS-CoV-2 positive newborns with early onset symptoms were reported from women with severe COVID-19, suggesting a possible viral transplacental transmission (5, 6). There is limited information for the association of COVID-19 and its direct complications to the growing fetus. In pregnant women, sever COVID-19 increases complications, particularly a higher incidence of iatrogenic pre-term delivery mostly due to fear of sudden maternal decompensation.

We report the case of a 34-year-old woman admitted to ICU due to severe COVID-19 infection whose newborn died by pulmonary embolism and thrombosis of the superior vena cava; immunohistochemical analysis demonstrated SARS-CoV-2 NP presence in neonatal tissues.

## Methods

Immunohistochemical analysis was performed on surgical specimens processed at the Anatomical Pathology Unit of the University of Ferrara. The samples analyzed were placenta, membranes, umbilical cord, lung, thrombus, small intestine, colon, stomach, kidney, liver, spleen, esophagus and heart. 4 μm tissue slides were stained for the detection of SARS-CoV-2 N-Protein (NP) (NB100-56576, Novus Biologicals, Centennial, 1:250 dilution) or Spike RBD (GTX635692, GeneTex, 1:100) and counterstained with Hematoxylin and Eosin for histo-morphological analysis. Immunohistochemical slide images were analyzed using QuPath software to calculate H-Score for SARS-CoV-2 and Spike RBD staining. A H-Score between 0 and 300 was obtained where 300 was equal to 100% of cells strongly stained (3+). H-Score comparisons were evaluated by Student *t*-test and *p*-values were corrected for multiple comparisons by Bonferroni’s correction. The statistical analysis was performed by GraphPad Software.

The study was conducted in accordance with the Declaration of Helsinki and the protocol was approved by the local Ethics Committee. The participant provided her written informed consent to this study.

## Case presentation

A 34-year-old pregnant woman, without history of diagnosed illnesses and not previously vaccinated against SARS-CoV-2, tested positive for SARS-CoV-2 with at the 23rd week of pregnancy. She was admitted to ICU with SARS-CoV-2 related pneumonia and underwent a cesarean section at 27 weeks due to her critical conditions. To notice, the woman did not have previous diagnosis of coagulation disorders, nor did we find any alterations in terms of routine blood coagulation profile.

The newborn was a male of 1,100 grams (90th percentile for 27 weeks, according to Intergrowth-21st tables for Very Preterm Infants) and he was given an Apgar index of 6 and 8 points at the first and fifth minute of life, respectively. The day of delivery the mother was tested positive again for SARS-CoV-2, while the newborn resulted negative. The laboratory infection diagnosis was performed through a pharyngeal swab and virus-specific RNA detection and amplification with real time polymerase chain reaction assays (RT-PCR).

The variant of SARS-CoV-2 that caused the infection has not been studied, but presumably it was the ‘‘English’’ variant (VOC202012/01, lineage B.1.1.7), which was responsible for 87% of the infections in Emilia Romagna region at that time.^1^

Placental samples did not show macroscopic parenchymal alterations. Multiple sections slide of parenchyma, umbilical cord and membranes were stained by standard hematoxylin-eosin-stained for histological evaluation, revealing only small foci of ischemic necrosis and inconspicuous fibrinous material in the intervillous space were found (Supplementary Figure 1). No signs of placentitis or any alteration of membranes and umbilical cord were demonstrated.

**FIGURE 1:**
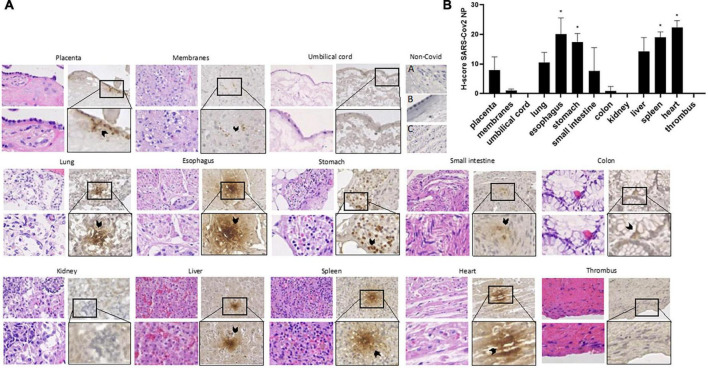
Immunohistochemical staining for SARS-CoV-2 N-Protein (NP) and Hematoxylin and Eosin staining for histo-morphological evaluation **(A)** and H-Score **(B)** for placental and neonatal tissues. Non-COVID-19 placenta (A), membranes (B) and umbilical cord (C) have been used as negative control for stain specificity. H-Score comparisons of the different tissues versus the placenta were evaluated by student *t*-test and *p*-values were corrected for multiple comparisons by Bonferroni’s correction. *p-value < 0.05. Magnification: 60× (upper line) and 100× (lower line).

The immunohistochemical examination showed positivity for SARS-CoV-2 NP in decidual placenta, but not in chorionic villi, and at lower extent in fetal membranes, at both amnion and chorion level (Figures 1A, B). On the contrary, we reported no SARS-CoV-2 NP positivity in umbilical cord (Figures 1A, B).

After cesarean, the infant was immediately transferred to the neonatal ICU, but deceased at the 37th day of life, due to progressive deterioration of the respiratory exchanges. Postmortem examination showed ectasia and congestion of the lung parenchyma with thrombosis of large and small pulmonary arteries, and thrombosis of the superior vena cava, without chronic thrombophilic-related diseases or iatrogenic factors. His tissues were analyzed and showed a different positivity for SARS-CoV-2 NP (Figures 1A, B).

SARS-CoV-2 NP was positive in the alveoli and bronchioles of lung tissue with a H-score similar to placental tissues and in the endocardium and myocardium of heart with a higher H-score compared to the placental tissue; kidney sections showed no inflammation and normal glomeruli, and were negative to for SARS-CoV-2 NP (Figure 1B, *p* < 0.01, *t*-student test).

We found positivity for SARS-CoV-2 NP in the gastrointestinal tract: the esophageal mucosa resulted positive in both esophageal glands and muscularis mucosa; the stomach presented positivity at lamina propria and muscolaris mucosae. Both organs presented a higher H-Score level compared to placenta (Figure 1B, *p* < 0.05, *t*-student test).

The small intestine showed positivity for SARS-CoV-2 NP in both mucosa and sub-mucosa, nearby the sub-mucosal glands; the colon showed a slight positivity in the crypt area but not in the submucosa. The liver showed positivity for SARS-CoV-2 NP in hepatocytes surrounding sinusoid microvascular structures, while the spleen was positive in white pulp, capsule, and red pulp, with a significant higher H-score compared to placenta (*p* < 0.05, *t*-student test). The thrombi were all negative for SARS-CoV-2 NP staining.

As a confirm, we stained the tissues for SARS-CoV-2 Spike protein, obtaining similar results, as reported in Supplementary Figure 2.

## Discussion

As for this case, the newborn death was caused by pulmonary embolism and thrombosis of the superior vena cava, a largely described event in COVID-19 patients.

Vertical viral transmission mother-to infant is generally known to occur at diverse levels: (I) intrauterine transmission, (II) during delivery, (III) during breast-feeding or (IV) by direct contact after delivery. In this report, the delivery was conducted through a cesarean section, the infant was not breastfed, and he was admitted to ICU immediately after birth. Since the infant never got out of the neonatal Intensive Care Unit, nor he came into contact with the mother or other subjects except from the ICU personnel, the opportunity of perinatal transmission was reduced, and the intrauterine transmission remains the most probable mode of infection of the infant.

Even if intrauterine vertical transmission from infected mothers to newborns remains a rare event, and most studies focusing on the presence of SARS-CoV-2 in placenta and blood cord, amniotic liquid have shown negative results, some reports suggest a possible intrauterine viral transmission (5–10).

In this case, negative nasopharyngeal tests do not exclude prior intrauterine infection: as Bwire et al. (11) stated in their meta-analysis concerning RT-PCR in different types of clinical specimens, the percentage of positivity to nasopharyngeal swabs is much lower (69.6%) than that observed for bronchoalveolar lavage fluid (91.8%) or rectal swabs (87.8%), showing how the intrinsic reliability of such tests should be somehow taken into consideration.

Kotlyar et al. (7) reviewed the literature to determine estimates of vertical transmission of SARS-CoV-2 based on early RNA detection: they selected a number of 38 cohort studies and 30 case reports concerning SARS-CoV-2 positive pregnant women, in which the viral RNA detection tests had been performed immediately at delivery or within 48 h after birth (for a total of 936 tested neonates included in the study). They found that nasopharyngeal swabs were positive in only 3.2% of neonates, concluding that vertical SARS-CoV-2 transmission was possible, but limited to a minority of cases. Successively, Moza et al. (10) have reviewed 75 reports with confirmed or possible vertical transmission and have described the adverse neonatal outcome: 32 cases of stillbirth (42.7%) and 24 symptomatic neonates (32%).

On the other hand, as a proof of concept of a possible vertical transmission, we observed SARS-CoV-2 NP and Spike protein positivity in different neonatal tissues. Neonatal intestinal tissues evidenced extent of SARS-CoV-2 NP positivity along the intestinal tract: such data are in line with recent results concerning SARS-CoV-2 bowel tropism (12, 13).

Interestingly, also spleen and liver, known to be organs strongly associated to the gastroenteric tract, present a consistent extent of SARS-CoV-2 NP and Spike protein positivity. This is supported by a case of a newborn tested positive for SARS-CoV-2 in both nasopharyngeal and rectal swabs taken after 1 h of life: the viral load at the rectal level was twice as high as that registered at the nasopharyngeal level. In that case, SARS-CoV-2 was also detected in placenta, amniotic fluid and maternal and newborn blood (5).

As discussed above, even if the newborn was tested negative for SARS-CoV-2 at birth, the contribution of the virus in the observed thrombosis could not be ignored, since we detected its presence in almost all the neonatal tissues analyzed. In our experience, we already showed how SARS-CoV-2 presence correlated with vascular damage and thrombosis in three patients tested negative for SARS-CoV-2 (12). Similarly, a 27 days-old newborn with pulmonary artery thrombosis associated with severe COVID-19 had tested negative for SARS-CoV-2 RT-PCR in nasopharyngeal swab, but positive in endotracheal aspirate (14).

Concerning this case, our hypothesis is that the augmented thrombophilic status induced by both premature birth and SARS-CoV-2 infection could have effectively played a significant role in the newborn thrombosis.

In preterm infants, hemorrhagic complications are more frequent than TE events in general, even if these ones can significantly increase neonatal morbidity and mortality. Chronic conditions, such as congenital nephrotic syndrome, iatrogenic complications (i.e., the placement of a central venous catheter) and bloodstream infections are described in the literature in association to neonatal deep venous thrombosis (15). The absence of chronic thrombophilic-related diseases or iatrogenic factors supports our hypothesis that the prothrombotic state could be at least partially caused by SARS-CoV-2 infection. This is similar to the findings described by Djordjevic et al. (8) who reported the cases of two children born from positive mothers who were tested positive for SARS-CoV-2 with elevated levels of viral specific IgM and elevated serum D-Dimer: both newborn showed signs of TE events and required ICU admissions in the first day after delivery.

## Conclusion

In SARS-CoV-2 positive pregnant women, intrauterine SARS-CoV-2 transmission might be considered as a possible cause of complications in the newborn, suggesting prompt diagnosis and therapy as beneficial for both maternal and newborn health. On the other hand, SARS-CoV-2 infection could induce thrombo-embolism in newborns similar to adult patients.

## Ethics statement

The studies involving human participants were reviewed and approved by the Local Ethics Committee (Comitato Etico di Area Vasta Emilia Centro della Regione Emilia-Romagna, CE-AVEC). Written informed consent to participate in this study was provided by patient’s mother. Written informed consent was obtained from the patient’s mother for the publication of this case report.

## Author contributions

SG and JMS contributed to the conception and design of the study and drafted the article. CS, CB, ES, and RG collected the biological samples and the clinical data. DB, RG, and RR contributed to the analysis and interpretation of data. PG, DB, RG, IM, RR, and AP revised it for important critical content. All authors gave their final approval of the version to be submitted.

## Footnotes

1
https://www.epicentro.iss.it/coronavirus/pdf/sars-cov-2-monitoraggio-varianti-indagini-rapide-18-marzo-2021.pdf

## Supplementary material

Supplementary Figure 1 Hematoxylin and Eosin staining for histo-morphological evaluation for placental tissues. Black arrow indicates small foci of ischemic necrosis. Magnification: 10x.

Supplementary Figure 2 Immunohistochemical staining for SARS-CoV-2 Spike RBD **(a)** and H-Score **(b)** for placental and neonatal tissues. H-Score comparisons of the different tissues versus the placenta were evaluated by Student *t*-test and *p*-values were corrected for multiple comparisons by Bonferroni’s correction. **p*-value < 0.05. Magnification: 100x.
