# Supplementary figures and images for: Case report: Tissue positivity for SARS-CoV-2 in a preterm born infant death of thrombosis: possible intrauterine transmission
Source: Front Med (Lausanne). 2023 May 11;10:1127529. doi: 10.3389/fmed.2023.1127529 (PMC10213910; doi:10.3389/fmed.2023.1127529)

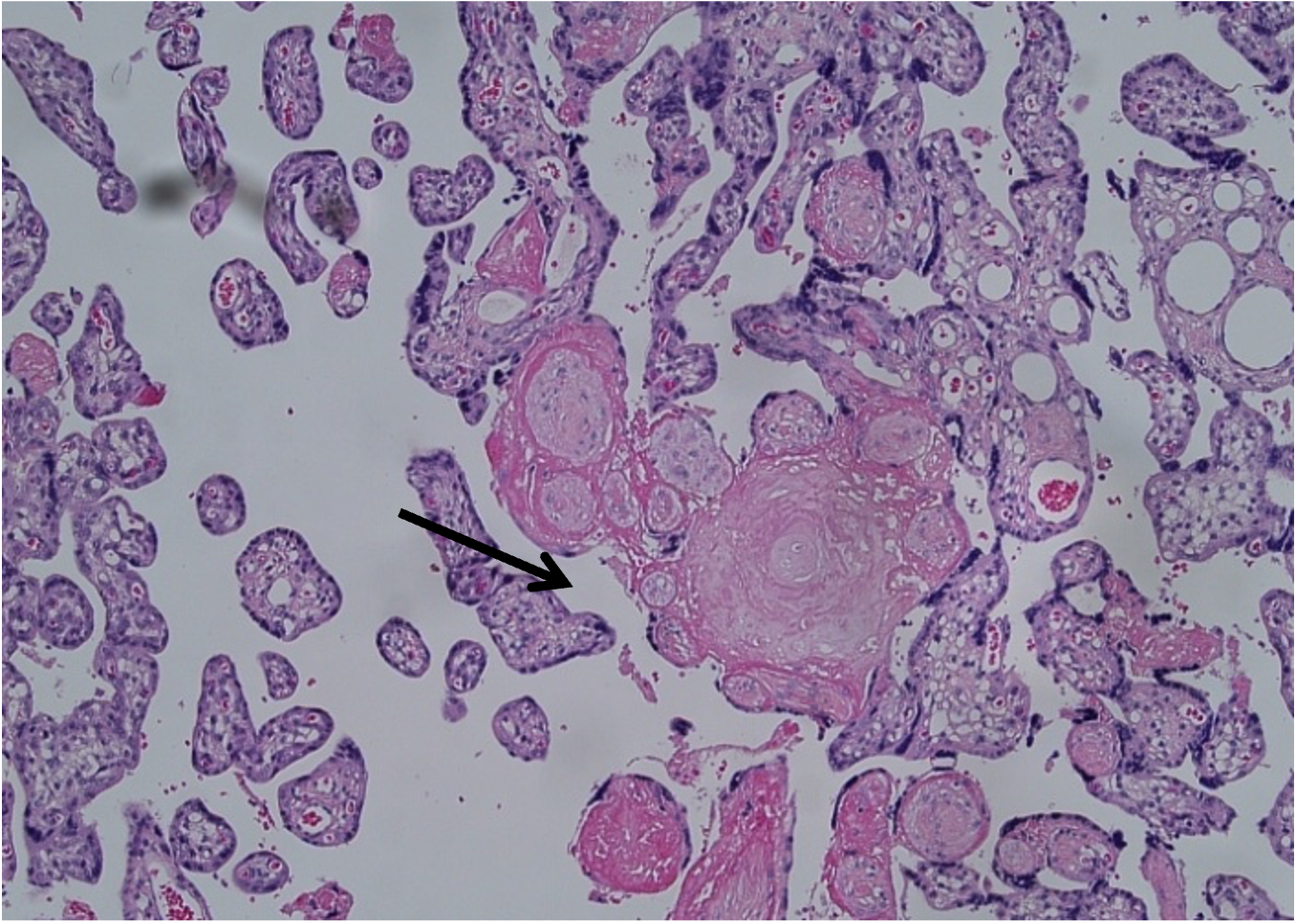

Supplement: Supplementary Figure 1 — Hematoxylin and Eosin staining for histo-morphological evaluation for placental tissues. Black arrow indicates small foci of ischemic necrosis. Magnification: 10x. [file Image_1.TIFF]

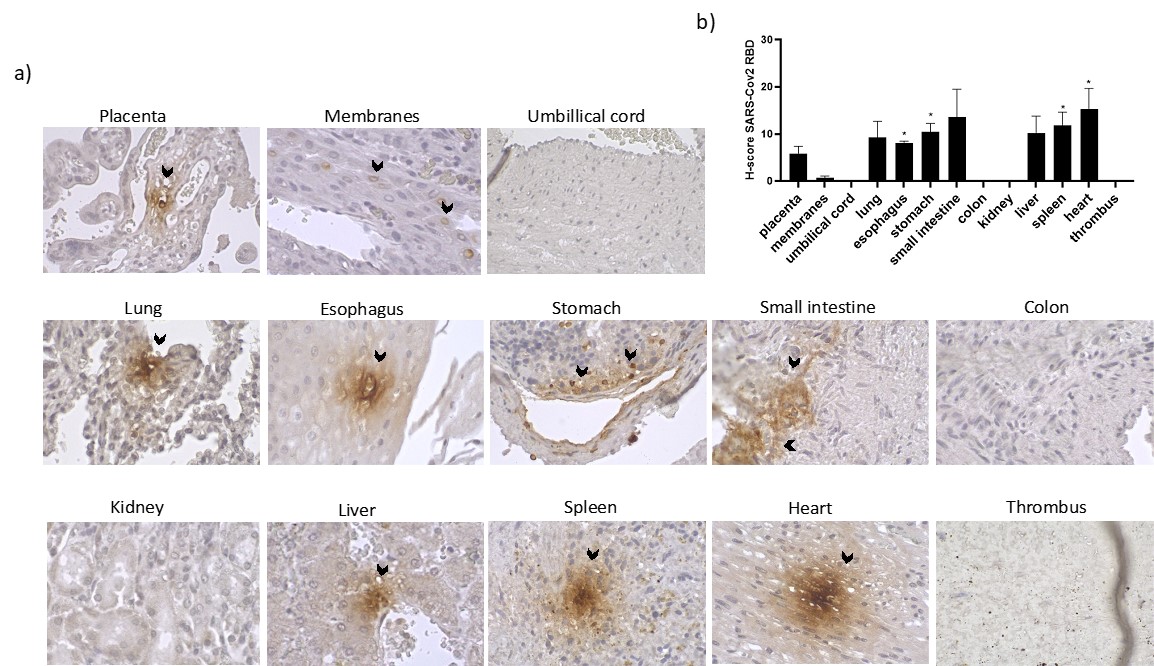

Supplement: Supplementary Figure 2 — Immunohistochemical staining for SARS-CoV-2 Spike RBD (a) and H-Score (b) for placental and neonatal tissues. H-Score comparisons of the different tissues versus the placenta were evaluated by Student t-test and p-values were corrected for multiple comparisons by Bonferroni’s correction. *p-value < 0.05. Magnification: 100x. [file Image_2.JPEG]
